# A Nationwide Exploration of Social Inequalities in Cancer Mortality Amidst the COVID‐19 Pandemic in Belgium

**DOI:** 10.1002/cam4.70487

**Published:** 2025-01-08

**Authors:** Yasmine Khan, Laura Van den Borre, Delphine De Smedt, Nick Verhaeghe, Brecht Devleesschauwer, Patrick Deboosere, Katrien Vanthomme, Sylvie Gadeyne

**Affiliations:** ^1^ Department of Public Health and Primary Care Ghent University Ghent Belgium; ^2^ Department of Epidemiology and Public Health, Sciensano Brussels Belgium; ^3^ Interface Demography, Department of Sociology Vrije Universiteit Brussel Brussels Belgium; ^4^ Department of Translational Physiology, Infectiology and Public Health Ghent University Merelbeke Belgium

**Keywords:** cancer, COVID‐19, mortality, social inequalities

## Abstract

**Background:**

The COVID‐19 pandemic disrupted global health systems, impacting cancer care and potentially increasing cancer mortality, especially among socioeconomically disadvantaged individuals. We aimed to assess changes in cancer mortality from March 1 to December 31, 2020 relative to the same period in 2019, and to examine potential shifts in cancer mortality's social disparities during the same time frame.

**Methods:**

We used nationwide individually linked cancer mortality data from the Belgian National Register, the Census 2011, and the tax register. Analyses were stratified by age group (45–59 years, 60–74 years, 75+ years) and sex across all cancer types, including breast, colorectal, lung, pancreatic, and prostate. Direct age‐standardized mortality rates were calculated in 2019 and 2020 to calculate absolute and relative changes in cancer mortality by social indicators. Relative inequalities in cancer mortality by social groups were calculated for both time frames using Poisson regression. Sensitivity analysis considered any mention of specified cancer groups on the Belgian death certificate.

**Results:**

For both overall and site‐specific cancers, our study found decreases in cancer mortality during the pandemic's early stages, particularly among individuals aged 75 and older. These changes did not significantly alter established socioeconomic patterns in cancer mortality.

**Conclusions:**

Reductions in reported cancer deaths in 2020 may reflect COVID‐19 prioritization in cause‐of‐death coding and its role as a competing risk, rather than true declines. Persistent educational disparities emphasize the need for continued policy and healthcare collaboration, with future research focused on the pandemic's long‐term effects on cancer mortality and social inequalities.

## Graphical abstract

We analyzed nationwide Belgian data to assess changes in cancer mortality during the early stages of the COVID‐19 pandemic (March–December 2020) compared to the same period in 2019, focusing on potential shifts in social disparities. Our findings showed decreases in reported cancer deaths, particularly among individuals aged 75 and older, without significant alterations in existing socioeconomic patterns. These reductions may reflect the prioritization of COVID‐19 in cause‐of‐death coding and its role as a competing risk, highlighting the need for continued policy and healthcare efforts to address persistent educational inequalities.

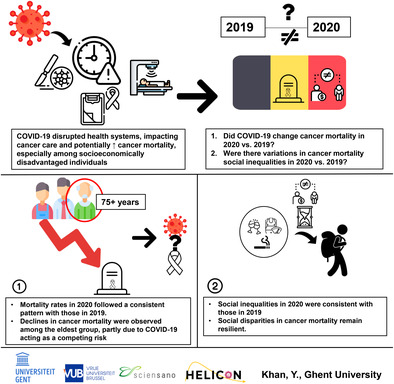

**Abbreviations**
ASMR: age‐standardized mortality rate
CI: confidence interval
COD: cause of death
ICD: International Statistical Classification of Diseases and Related Health Problems
ISCED: International Standard Classification of Education
MRR: mortality rate ratio

## 1 Introduction

Despite advancements in cancer screening, diagnosis, and treatment, cancer remains a major global health concern, accounting for 10 million deaths in 2019, making it the second leading cause of death (COD) worldwide [1]. The economic burden of cancer in Europe now exceeds €100 billion annually, according to the Europe's Beating Cancer Plan [2]. An important factor influencing cancer dynamics is the socioeconomic status of the patient. Individuals with deprived socioeconomic status, as measured by factors such as education, income, or other indicators, face barriers to receiving care, increased comorbidities, and higher rates of early cancer mortality [3, 4]. Over time, the pertinence of these social disparities has been increasingly accentuated in numerous international studies, emphasizing the need for rigorous measurement and long‐term monitoring [5].

The early months of the COVID‐19 pandemic placed immense pressure on healthcare systems worldwide [6]. Due to the surge in acute COVID‐19 hospitalizations, the provision of care for non‐COVID‐19 conditions, including cancer, was disrupted. Although delays in treatment have been associated with increased mortality risk, the effects of the pandemic on cancer mortality are likely multifaceted [7, 8, 9, 10, 11]. Beyond delays, the crisis introduced competing mortality risks, with COVID‐19 becoming a common COD and changes in death certificate coding prioritizing COVID‐19, as per WHO guidelines [12]. Additionally, many cancer patients, already vulnerable due to weakened immune systems, may have avoided seeking medical care out of fear of exposure, exacerbating delays in treatment [13]. Research on cancer mortality during the pandemic has shown mixed results; some reported declines due to COVID‐19 being recorded as the underlying COD, possibly underestimating cancer deaths [10, 11]. On the other hand, research that considered multiple causes noted increases in cancer mortality, underscoring the complexity of the pandemic's impact [8, 9]. These combined factors contribute to the challenge of accurately assessing the pandemic's true effect on cancer mortality.

Increased social disparities in cancer mortality could be expected, as individuals from lower socioeconomic backgrounds, already burdened by comorbidities and limited healthcare access, likely faced greater risks from delays in care and competing causes of death like COVID‐19 [14]. Higher COVID‐19 mortality in these groups could shift mortality patterns, masking true cancer mortality inequalities. Additionally, fear of infection during the pandemic was more pronounced in lower socioeconomic groups, who often had less access to reliable information and resources, further discouraging timely care‐seeking and exacerbating their health outcomes [15, 16]. A study from the Netherlands found increased income‐related inequalities in cancer deaths during 2020, highlighting the pandemic's potential impact on exacerbating social disparities in cancer mortality [17].

In light of these findings, the limited literature, and the inconsistency of earlier research results on the pandemic's impact on social inequalities in cancer mortality, this study uniquely assesses both the overall impact of the early phase of the COVID‐19 pandemic on cancer mortality and associated social inequalities in Belgium. Specifically, we will examine changes in cancer mortality between March 1, 2020 and December 31, 2020 compared to the same time frame in 2019 (the pre‐COVID‐19 reference period). Additionally, we will evaluate socioeconomic disparities in cancer mortality in 2020 compare to those in 2019.

## 2 Materials and Methods

### 2.1 Dataset and Study Population

In this nationwide retrospective population‐based study, we used comprehensive and pseudonymized cause‐specific mortality data (cancer mortality data) of 2019 and 2020 for the total population legally residing in Belgium aged 45 years and above. The data were derived from Statistics Belgium and involved individually linked data from various administrative data sources containing a wide range of socioeconomic and sociodemographic variables [18]. These sources included the Belgian National Register, which provided annual population stocks containing information on sociodemographic characteristics such as living arrangement, mortality data for all individuals officially residing in Belgium on January 1 of each year [19], and COD data originating from the death certificates. Additionally, the administrative census of 2011 provided education data, whereas the tax register (IPCAL database containing the most recent information up to 2017) supplied yearly personal income data.

### 2.2 Variables

#### 2.2.1 Selection of Cancer Types

To identify cancer deaths, we used the International Statistical Classification of Diseases and Related Health Problems (ICD), Tenth Revision. Analyses were conducted for overall malignant cancer sites, going from C00 to C97, and for five site‐specific cancers: breast (C50, for women only), colorectal (C18–C20), lung (C33–C34), pancreatic (C25), and prostate (C61, for men only) cancers. Breast, colorectal, lung, and prostate cancers were selected, as they were the most prevalent cancers for both men and women in 2019 in Belgium [20]. Pancreatic cancer was selected as it is ranked third among cancers with the highest mortality rates among men in Belgium, which illustrates its aggressiveness [20, 21].

#### 2.2.2 Sociodemographic and Socioeconomic Variables

To obtain a comprehensive understanding of social disparities in cancer mortality, we focused on educational level and income as socioeconomic indicators and living arrangement as a sociodemographic indicator [22, 23]. The education variable was categorized using the International Standard Classification of Education (ISCED) 2011 framework [24]. We distinguished primary or less education (ISCED 0–1), lower secondary education (ISCED 2), upper secondary education (ISCED 3–4), and higher education (ISCED 5–6). Income represents economic capacity, with higher deciles often enjoying improved living conditions and healthcare access, which are critical for health [22]. Income was categorized into deciles based on net taxable income per person, which refers to income before tax, after social security contributions have been paid and costs deducted. We distinguished people with a high income (decile eight to 10), a middle income (five to seven), a low income (deciles one to four), and no or undeclared income (zero decile). The zero decile category consists of a diverse group of individuals with varying economic backgrounds. This group may include wealthier individuals for instance, employees of the European Commission, people living off rental income, and students who rely on financial support from their parents for daily expenses. Conversely, it can also include individuals with no income sources, who are considered poor. Currently, there is no method to determine the predominant income level within this group—whether it is the wealthier or the poorer members. For completeness, we included the zero decile group in our analyses but did not interpret their results due to their heterogeneity. Household living arrangements give information on one's social context, with living alone potentially limiting support and shared households providing emotional benefits but also potential risks of infectious disease outbreaks [22]. The latter variable differentiated individuals living with a partner, those living without a partner, other scenarios (children living with parents, multigenerational families), and collective households (including elderly care homes, specialized residential care homes, and prisons). Missing values, which might be unevenly distributed, were treated as separate categories for all three social indicators.

### 2.3 Statistical Analysis

To answer the first research question related to changes in cancer mortality during the COVID‐19 pandemic, we used direct standardization and calculated age‐standardized mortality rates (ASMRs) and 95% CI. The ASMRs were calculated for the period March 1, 2020—December 31, 2020 and compared to those calculated for the same time frame in 2019. The total Belgian population on January 1, 2020 served as the reference population for direct standardization. Absolute differences and relative changes (ratio between the absolute difference and the 2019 AMSR multiplied by 100) between both series of ASMRs were calculated by socioeconomic and sociodemographic indicators, first for all cancers (C00–C97) and then by type of cancer. All analyses were stratified by sex and three age categories (aged 45–59, 60–74, and 75 and over). The significance of the mortality difference between 2019 and 2020 was formally tested for both sexes and each age group as explained by Altman and Bland [25].

To answer our second research question related to changes in the social inequalities in cancer mortality in 2020 compared to those in 2019, we estimated relative inequalities using Poisson regression analyses for all cancer types combined, and for breast, colorectal, lung, pancreatic, and prostate cancers in 2019 and 2020. All analyses were stratified by sex and three age categories. Age‐adjusted (treated as a continuous variable) cancer mortality rate ratios (MRRs) and 95% CI were calculated by education, income, and living arrangement with higher education, high income, and living with a partner as respective reference categories. We focused on relative inequality to facilitate comparisons across different groups and highlight proportional differences. However, relative measures are influenced by the overall level of mortality, with lower mortality potentially showing higher relative inequalities. They also do not capture the absolute burden (i.e., the actual number of deaths), which is crucial for understanding the real‐world impact. To address this, we included absolute differences in cancer mortality in Table 2 (see Section 3) to provide a comprehensive view of both proportional and absolute impacts. In our fully adjusted model, we controlled for the different socioeconomic and sociodemographic variables simultaneously to account for their potential confounding effects on the relationship between social factors and cancer mortality. The log of the person‐time was used as the offset. The person‐time was calculated for each participant from their entry into the study (February 28, 2019 and February 29, 2020) until their exit due to the cancer mortality or the end of the study (December 31, 2019 or 2020). Again, the significance of the cancer mortality differences between the pre‐COVID‐19 and the COVID‐19 was formally tested as explained by Altman and Bland [25]. Preliminary correlation analyses between the different social variables were carried out to check for overdispersion and multicollinearity. Although there were correlations present among the variables, they were not of a magnitude that would compromise the validity of our findings. All analyses were performed with Stata/SE 18.0.

#### 2.3.1 Sensitivity Analysis

To test potential challenges in correctly coding the COD, we conducted a sensitivity analysis. Instead of focusing solely on the primary COD (the initial condition that led to the sequence of health issues culminating in death), we expanded our analysis to include all mentions of these cancers in the causal chain that is mentioned on the Belgian death certificate. This chain encompasses seven distinct sections: the immediate COD (the final health condition causing death), three levels of contributing causes (which may include conditions that led to the immediate cause or significant health issues contributing to death), and additional relevant health conditions that, while not directly leading to death, were present and significant.

## 3 Results

Table 1 provides the sociodemographic and socioeconomic characteristics of the study population, offering context for subsequent analyses on cancer mortality changes and social disparities.

**TABLE 1:** Characteristics of the study population: Number of cancer patients by age group (45–59, 60–74, 75+ years), year (2019 and 2020), sex, cancer site, and socioeconomic dimensions.

	45–59		60–74		75+	
	2020	2019	2020	2019	2020	2019
*Women*						
Total	974,638	977,386	804,777	779,819	461,225	419,716
Educational level						
Primary or less	65,714	70,789	137,616	140,938	168,400	156,634
Lower secondary	146,631	155,942	216,333	213,275	123,523	111,635
Upper secondary	323,666	324,976	210,720	198,272	68,929	61,371
Higher education	330,163	324,649	182,849	171,880	53,776	46,997
Missing	108,462	101,031	57,260	55,454	46,595	43,080
Income level						
Zero decile	34,746	34,835	17,447	16,134	4629	4291
Low	360,731	370,994	419,190	413,490	253,011	229,252
Middle	222,414	223,182	206,331	203,050	149,718	138,278
High	332,763	329,633	154,674	141,683	51,556	46,194
Missing	23,984	18,741	7135	5461	2309	1702
Living arrangement						
With partner	661,157	661,635	513,443	492,289	162,563	140,907
Without partner	273,506	276,244	254,858	251,886	234,324	218,051
Other	37,156	36,503	29,866	28,803	21,867	20,354
Collective households	2816	3004	6611	6841	42,468	40,405
*Men*						
Total	989,233	988,504	745,681	715,382	300,860	266,529
Educational level						
Primary or less	71,200	75,192	112,609	112,667	90,759	82,937
Lower secondary	178,461	186,623	193,494	186,582	72,497	63,651
Upper secondary	335,107	332,472	199,052	187,249	53,270	46,019
Higher education	269,770	268,341	182,824	174,581	58,021	50,193
Missing	134,695	125,875	57,699	54,301	26,313	23,728
Income level						
Zero decile	34,569	34,702	12,093	10,827	1893	1746
Low	184,845	189,048	172,447	167,441	105,084	95,865
Middle	211,387	213,832	306,549	308,566	142,350	124,351
High	527,829	527,458	246,942	222,893	50,351	43,802
Missing	30,604	23,463	7650	5654	1181	765
Living arrangement						
With partner	683,193	685,278	556,222	536,314	208,645	182,896
Without partner	234,865	235,108	157,188	149,069	73,038	66,304
Other	65,671	62,504	25,278	23,196	8093	7290
Collective households	5504	5613	6993	6802	11,084	10,039

### 3.1 Change in Cancer Mortality in 2020 Compared to That in 2019

This section aims to compare cancer mortality during the COVID‐19 and the pre‐COVID‐19 period, using absolute and relative ASMR differences. Both overall cancer deaths and the five selected cancer types are considered.

#### 3.1.1 All Cancers (C00–C97)

Table 2 shows the absolute differences between cancer mortality in 2019 and cancer mortality in 2020, and the relative change in the different subpopulations by socioeconomic and sociodemographic indicators. Our results showed that there was a general decrease in cancer mortality across both sexes and the three age groups, especially pronounced among men and women aged 75 years or over. There was an exception to this trend, with men aged 45–59 years with upper secondary education who experienced a significant 14% increase in cancer mortality.

**TABLE 2:** Absolute cancer mortality differences per 100,000 person‐years with 95% confidence interval (95% CI) and relative change (%) in age‐standardized mortality rates (ASMRs) for all cancers in 2020 compared to those in 2019 for women and men by age group, and by socioeconomic and sociodemographic indicators.

	45–59 years		60–74 years		75 years or over	
	Absolute	%	Absolute	%	Absolute	%
*Women*						
Total	**−10.80**	**−7.46**	−15.60	−3.13	**−353.00**	**−16.82**
Educational level						
Primary or less	8.30	3.98	**−58.20**	**−9.85**	**−488.00**	**−21.61**
Lower secondary	−8.30	−4.58	0.90	0.17	−205.20	−10.59
Upper secondary	−16.90	−11.97	0.40	0.09	−217.30	−11.16
Higher education	−14.00	−12.07	−18.70	−4.57	**−393.40**	**−19.04**
Missing	8.90	6.34	−16.70	−3.12	**−318.70**	**−15.34**
Income level						
Zero decile	−27.30	−9.73	**−229.40**	**−35.31**	600.60	87.97
Low	−10.40	−5.77	−20.40	−3.91	**−372.10**	**−17.89**
Middle	5.90	4.74	9.90	1.96	**−377.20**	**−17.47**
High	**−20.70**	**−19.85**	−14.40	−3.66	−240.50	−11.62
Missing	8.30	6.99	229.40	50.00	−509.20	−23.54
Living arrangement						
With partner	**−11.80**	**−9.49**	−12.80	−2.98	**−483.60**	**−25.18**
Without partner	−0.60	−0.34	−12.40	−2.10	**−286.30**	**−14.64**
Other	**−71.60**	**−32.11**	−45.00	−7.47	**−420.50**	**−19.45**
Collective households	119.50	22.71	−133.70	−9.15	**−309.70**	**−11.11**
*Men*						
Total	−0.80	−0.53	**−51.00**	**−6.40**	**−815.80**	**−19.64**
Educational level						
Primary or less	−40.50	−14.12	−62.80	−5.84	**−888.10**	**−19.73**
Lower secondary	4.30	2.37	−47.80	−5.35	**−1036.60**	**−23.95**
Upper secondary	**18.80**	**13.65**	**−64.50**	**−8.68**	**−593.80**	**−15.37**
Higher education	−8.80	−9.08	−19.90	−3.67	**−707.60**	**−19.68**
Missing	−6.50	−4.35	−85.90	−9.37	**−552.40**	**−14.19**
Income level						
Zero decile	39.10	14.14	−43.30	−5.23	−486.80	−23.28
Low	−11.20	−4.53	**−106.30**	**−9.51**	**−1012.20**	**−23.23**
Middle	−16.20	−9.06	−31.90	−3.98	**−725.30**	**−17.39**
High	9.80	10.28	−29.40	−5.39	**−595.70**	**−16.03**
Missing	−5.80	−4.37	185.40	28.47	−930.20	−25.33
Living arrangement						
With partner	−3.20	−2.55	**−50.00**	**−7.05**	**−872.90**	**−21.70**
Without partner	3.80	1.86	**−75.30**	**−7.24**	**−509.30**	**−12.66**
Other	−10.60	−5.52	120.70	16.80	**−921.30**	**−23.54**
Collective households	152.70	26.21	−153.60	−5.77	**−1380.60**	**−21.13**

*Note:* Values in bold indicate a significant difference (*p* < 0.05) in ASMRs between 2020 and 2019.

#### 3.1.2 Site‐Specific Cancers

The oldest age group (75 years or over) showed the most significant declines in mortality for all five cancer types in 2020 compared to that in 2019: a 19% decline for breast cancer in women (Table 3); an 18% and 17% decline for colorectal cancer in, respectively, women and men (Table 4); a 15% and 21% decline for lung cancer in, respectively, women and men (Table 5); a 23% decline for pancreatic cancer in men (Table 6); and a 17% decline for prostate cancer in men (Table 7). Some younger age groups also showed declines that were similar to those in the oldest age group, although not consistent across all cancer types. For example, breast cancer mortality among women aged 45–59 declined by 16% (Table 3) and colorectal cancer mortality among women aged 60–74 decreased by 15% (Table 4). Men aged 60–74 years faced a significant 9% reduction in lung cancer mortality (Table 5).

**TABLE 3:** Absolute cancer mortality differences per 100,000 person‐years with 95% confidence interval (95% CI) and relative change (%) in age‐standardized mortality rates (ASMRs) for breast cancer in 2020 compared to those in 2019 for women by age group, and by socioeconomic and sociodemographic indicators.

	45–59 years		60–74 years		75 years or over	
	Absolute	%	Absolute	%	Absolute	%
*Women*						
Total	**−5.40**	**−15.88**	1.80	2.31	**−70.10**	**−18.83**
Educational level						
Primary or less	0.20	0.66	−9.50	−11.39	**−97.40**	**−25.92**
Lower secondary	−12.60	28.44	9.00	12.10	−53.20	−15.43
Upper secondary	−2.80	−9.30	13.90	19.77	42.70	12.49
Higher education	−7.50	−21.99	−4.30	−5.31	−76.60	−17.55
Missing	−3.30	−9.94	−26.50	−28.13	**−135.60**	**−32.94**
Income level						
Zero decile	−14.50	−27.67	−51.70	−46.96	−26.10	−25.39
Low	**−10.80**	**−24.88**	6.90	9.09	**−71.20**	**−18.98**
Middle	1.40	4.84	5.20	6.62	**−77.90**	**−21.32**
High	−2.60	−10.44	−17.20	−21.86	−39.30	−9.70
Missing	−6.40	−15.96	33.20	37.01	106.80	27.85
Living arrangement						
With partner	**−6.20**	**−20.20**	−0.20	−0.29	−60.70	−19.00
Without partner	−7.00	−16.71	0.60	0.69	**−68.20**	**−21.52**
Other	23.90	87.23	18.40	21.15	−24.20	−8.51
Collective households	−41.80	−34.98	14.20	6.18	−11.67	56.55

*Note:* Values in bold indicate a significant difference (*p* < 0.05) in ASMRs between 2020 and 2019.

**TABLE 4:** Absolute cancer mortality differences per 100,000 person‐years with 95% confidence interval (95% CI) and relative change (%) in age‐standardized mortality rates (ASMRs) for colorectal cancer in 2020 compared to those in 2019 for women and men by age group, and by socioeconomic and sociodemographic indicators.

	45–59 years		60–74 years		75 years or over	
	Absolute	%	Absolute	%	Absolute	%
*Women*						
Total	−2.50	−23.15	**−6.80**	**−14.91**	**−54.50**	**−18.29**
Educational level						
Primary or less	−5.20	−37.14	−5.00	−9.90	**−78.10**	**−23.83**
Lower secondary	−4.20	−34.15	−2.30	−5.18	1.70	0.67
Upper secondary	−1.90	−19.00	0.30	0.80	−64.20	−21.69
Higher education	−2.10	−23.33	−12.30	−27.64	−114.50	−33.01
Missing	−0.60	−4.48	−26.30	−38.79	−39.50	−14.93
Income level						
Zero decile	−8.30	−28.62	−7.90	−14.31	64.00	35.71
Low	−2.60	−20.47	**−10.90**	**−23.00**	−43.90	−15.73
Middle	**−7.80**	**−63.41**	−1.80	−4.13	**−63.30**	**−19.82**
High	1.90	36.54	4.20	10.24	−75.00	−23.76
Missing	−0.40	−3.17	−19.50	−30.71	−37.20	−19.40
Living arrangement						
With partner	−1.20	−16.44	−7.30	−18.02	−23.10	−9.38
Without partner	3.80	44.19	−3.90	−7.46	**−50.20**	**−17.83**
Other	−8.80	−52.07	**−39.50**	**−56.75**	−19.30	−6.15
Collective households	56.00	74.47	44.00	82.71	**−92.70**	**−22.91**
*Men*						
Total	−1.00	−7.35	−4.70	−7.00	**−78.80**	**−17.35**
Educational level						
Primary or less	0.90	3.78	−4.70	−5.29	**−118.40**	**−23.04**
Lower secondary	−1.30	−10.66	−6.90	−9.54	**−196.40**	**−36.22**
Upper secondary	0.20	1.39	−1.40	−2.32	−26.00	−6.72
Higher education	−3.60	−26.28	−2.60	−4.93	115.30	36.88
Missing	0.90	18.00	−13.70	−18.36	−51.30	−13.83
Income level						
Zero decile	0.10	0.56	43.00	147.26	−343.00	−47.94
Low	−2.00	−10.53	−9.20	−10.54	**−126.50**	**−25.72**
Middle	**−7.40**	**−36.10**	−6.50	−9.83	−60.30	−13.75
High	1.80	20.93	−1.50	−2.59	−14.40	−3.58
Missing	7.10	215.15	−28.20	−30.10	646.60	1010.31
Living arrangement						
With partner	−1.20	−9.76	−6.80	−11.47	−69.30	−16.53
Without partner	−4.10	−24.26	10.10	11.43	−95.40	−19.07
Other	9.10	61.49	−21.80	−34.88	−37.20	−14.35
Collective households	30.80	198.71	−81.70	−36.62	−94.00	−14.88

*Note:* Values in bold indicate a significant difference (*p* < 0.05) in ASMRs between 2020 and 2019.

**TABLE 5:** Absolute cancer mortality differences per 100,000 person‐years with 95% confidence interval (95% CI) and relative change (%) in age‐standardized mortality rates (ASMRs) for lung cancer in 2020 compared to those in 2019 for women and men by age group, and by socioeconomic and sociodemographic indicators.

	45–59 years		60–74 years		75 years or over	
	Absolute	%	Absolute	%	Absolute	%
*Women*						
Total	0.10	0.32	−4.30	3.47	**−27.70**	**−15.45**
Educational level						
Primary or less	10.60	18.86	−9.90	−5.85	**−45.20**	**−23.04**
Lower secondary	2.50	5.56	−1.30	−0.89	−13.10	−7.18
Upper secondary	−2.60	−8.00	−0.50	−0.46	−22.80	−15.00
Higher education	2.60	19.12	3.50	5.31	−29.90	−16.00
Missing	1.50	4.62	−16.20	−11.16	−3.00	−2.03
Income level						
Zero decile	−12.20	−16.14	−21.80	−19.75	−0.70	−5.11
Low	2.60	6.27	−4.30	−3.15	**−36.40**	**−21.10**
Middle	−3.50	−10.45	−7.50	−5.55	−25.30	−13.38
High	1.40	11.20	2.80	4.12	6.40	3.51
Missing	15.90	138.26	20.20	13.58	−112.20	−30.71
Living arrangement						
With partner	0.10	0.43	−2.00	−2.01	**−60.50**	**−37.28**
Without partner	3.70	8.19	−9.70	−5.97	−22.30	−11.90
Other	**−27.90**	**−56.25**	11.50	8.24	26.50	20.43
Collective households	41.10	39.98	33.00	9.58	−25.20	−12.14
*Men*						
Total	−3.70	−8.62	**−22.40**	**−8.88**	**−154.50**	**−20.78**
Educational level						
Primary or less	**−35.20**	**−29.81**	−31.00	−7.66	**−186.30**	**−20.33**
Lower secondary	0.20	0.37	−19.50	−6.55	−209.60	−27.00
Upper secondary	6.00	17.29	**−33.40**	**−15.31**	−62.10	−10.50
Higher education	−5.30	−31.55	−14.50	−11.19	−46.30	−10.66
Missing	−6.40	−13.17	3.90	1.35	−215.10	−26.64
Income level						
Zero decile	1.50	1.82	58.10	23.31	−185.80	−83.81
Low	−14.10	−16.61	−20.40	−5.47	**−252.20**	**−28.17**
Middle	−3.00	−5.80	−20.60	−7.81	−95.50	−13.40
High	0.60	2.87	**−28.00**	**−19.49**	−82.60	−16.63
Missing	−4.40	−10.50	151.10	91.91	251.80	136.11
Living arrangement						
With partner	−2.40	−7.62	**−26.20**	**−11.81**	**−153.50**	**−20.55**
Without partner	−3.70	−5.57	−29.50	−8.63	−121.00	−16.20
Other	**−29.10**	**−43.83**	97.00	45.93	44.40	6.44
Collective households	87.60	40.78	15.10	1.85	**−338.90**	**−36.97**

*Note:* Values in bold indicate a significant difference (*p* < 0.05) in ASMRs between 2020 and 2019.

**TABLE 6:** Absolute cancer mortality differences per 100,000 person‐years with 95% confidence interval (95% CI) and relative change (%) in age‐standardized mortality rates (ASMRs) for pancreatic cancer in 2020 compared to those in 2019 for women and men by age group, and by socioeconomic and sociodemographic indicators.

	45–59 years		60–74 years		75 years or over	
	Absolute	%	Absolute	%	Absolute	%
*Women*						
Total	−1.30	−14.29	−2.40	−5.62	−13.80	−9.06
Educational level						
Primary or less	7.10	116.39	**−15.60**	**−33.69**	−16.00	−10.26
Lower secondary	1.10	8.59	1.20	2.57	−10.00	−6.81
Upper secondary	−4.20	−36.21	−5.60	−11.86	−27.90	−18.32
Higher education	−1.20	−18.75	4.00	12.08	24.60	21.10
Missing	−3.00	−32.26	2.40	6.78	−21.90	−12.52
Income level						
Zero decile	1.70	9.55	**−63.20**	**−74.00**	−92.10	−87.63
Low	−1.60	−14.68	**−9.40**	**−19.50**	−29.90	−19.22
Middle	2.20	37.29	7.30	19.73	2.00	1.37
High	**−4.50**	**−48.39**	7.40	21.70	−4.50	−2.61
Missing	0.00	0.00	31.40	147.42	−9.10	−25.14
Living arrangement						
With partner	−2.20	−23.66	−1.10	−2.84	−60.40	−31.86
Without partner	1.50	19.74	−4.80	−9.54	−8.60	−5.48
Other	−8.40	−50.91	−4.80	−12.53	−13.80	−10.49
Collective households	25.20	98.05	16.30	23.66	8.20	6.68
*Men*						
Total	1.80	16.36	1.30	2.50	**−41.00**	**−22.59**
Educational level						
Primary or less	6.10	58.10	−8.20	−12.50	−23.70	−14.64
Lower secondary	4.80	44.86	6.80	12.76	−12.70	−6.97
Upper secondary	4.70	43.52	9.20	20.35	−61.90	−34.91
Higher education	−4.80	−39.02	−2.40	−5.04	**−107.40**	**−46.29**
Missing	0.10	1.09	−11.50	−20.39	−36.90	−20.30
Income level						
Zero decile	3.20	38.10	**73.50**	**680.56**	177.30	—
Low	2.80	19.58	−9.30	−15.37	−30.40	−16.68
Middle	0.10	0.89	7.90	15.58	−24.60	−15.95
High	2.80	29.17	−0.20	−0.43	**−112.00**	**−44.03**
Missing	−7.10	−60.68	10.50	19.02	−476.30	−85.39
Living arrangement						
With partner	−0.70	−6.14	1.70	3.44	−23.30	−11.83
Without partner	**8.90**	**86.41**	−0.10	−0.16	−48.80	−24.77
Other	5.80	74.36	11.00	29.33	−135.40	−57.74
Collective households	−15.50	−100.00	−31.60	−31.29	−68.70	−35.10

*Note:* Values in bold indicate a significant difference (*p* < 0.05) in ASMRs between 2020 and 2019.

**TABLE 7:** Absolute cancer mortality differences per 100,000 person‐years with 95% confidence interval (95% CI) and relative change (%) in age‐standardized mortality rates (ASMRs) for prostate cancer in 2020 compared to those in 2019 for women and men by age group, and by socioeconomic and sociodemographic indicators.

	45–59 years		60–74 years		75 years or over	
	Absolute	%	Absolute	%	Absolute	%
*Men*						
Total	0.80	22.86	−6.40	−12.28	**−146.90**	**−16.69**
Educational level						
Primary or less	4.50	214.29	−5.10	−9.77	−107.40	−12.38
Lower secondary	0.00	0.00	−7.20	−12.02	−134.10	−15.62
Upper secondary	−0.30	−10.34	−7.40	−14.54	−183.80	−19.37
Higher education	2.00	74.07	−8.60	−19.95	**−232.40**	**−23.96**
Missing	1.30	22.81	−0.10	−0.16	−135.10	−18.17
Income level						
Zero decile	−0.10	−1.03	7.90	21.41	545.30	3760.69
Low	−1.00	−24.39	−9.70	−15.16	**−140.80**	**−16.26**
Middle	0.00	0.00	−3.50	−6.63	**−163.50**	**−18.02**
High	1.70	68.00	−9.50	−23.17	−121.60	−13.99
Missing	6.00	181.82	63.10	195.96	−1517.70	−94.30
Living arrangement						
With partner	1.00	29.41	−6.10	−13.01	−99.40	−13.24
Without partner	−0.50	−10.42	−6.70	−10.98	**−159.70**	**−18.76**
Other	3.70	0.00	2.30	3.38	−47.60	−5.38
Collective households	0.00	0.00	−33.50	−16.33	−316.30	−17.35

*Note:* Values in bold indicate a significant difference (*p* < 0.05) in ASMRs between 2020 and 2019.

#### 3.1.3 Sensitivity Analysis

Our sensitivity analysis yielded results consistent with our initial findings, indicating that individuals aged 75 and older experienced significant decreases in cancer mortality in 2020 compared to those in 2019, across all cancers and the site‐specific cancers (Data S1).

### 3.2 Changes in Social Disparities in 2020 Compared to Those in 2019

This section aims to assess shifts in social inequalities in cancer mortality, focusing primarily on education. Detailed results for income‐related and living arrangement‐related disparities can be found in Data S1.

#### 3.2.1 All Cancers

**FIGURE 1:**
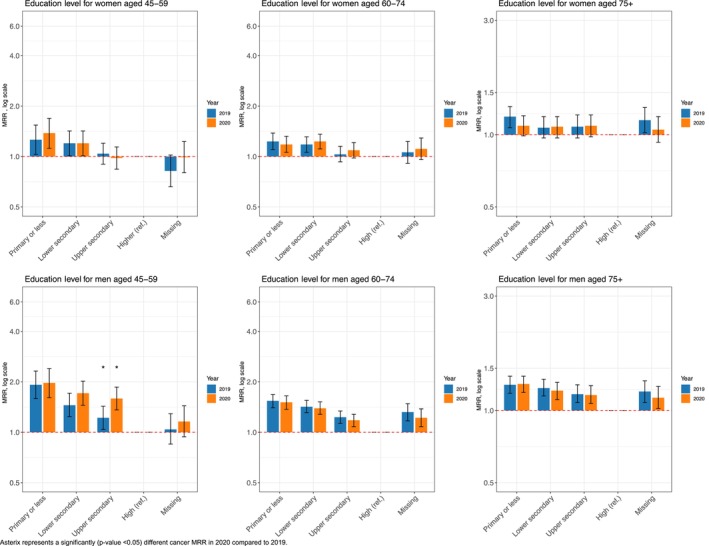
Education‐related cancer mortality rate ratios (MRRs) and 95% CI for all cancers (C00–C97) in 2020 and 2019, among the 45–59, 60–74, and 75+ age groups, by sex.

Overall, our fully adjusted Poisson regression models revealed minimal significant changes in social inequalities in cancer mortality in 2020 compared to those in 2019. Individuals with lower education consistently faced increased cancer mortality rates compared to their counterparts with higher education. In 2020, men aged 45–59 years with upper secondary education had a 59% higher risk of cancer mortality compared to men with higher education (MRR in 2020: 1.59; 95% CI: 1.36, 1.86) (Figure 1). These educational disparities persisted for both men and women in the 45–59 and 60–74 age groups. However, among the oldest individuals, only men showed consistent educational disparities, whereas no noticeable differences by education were found for women (Figure 1).

#### 3.2.2 Site‐Specific Cancers

In general, our fully adjusted Poisson regression models showed a consistent pattern of social inequalities in cancer mortality in 2020 compared to that in 2019 for all five selected cancer types. When focusing on education‐related disparities, we observed that particularly men in the 45–59‐year group and women in the 60–74‐year group had an increased relative risk of death in 2020 compared to those in 2019 for colorectal, lung, and pancreatic cancer mortality. In contrast, no significant changes in relative risks were noted for breast cancer in women and prostate cancer in men.

#### 3.2.3 Breast Cancer

**FIGURE 2:**
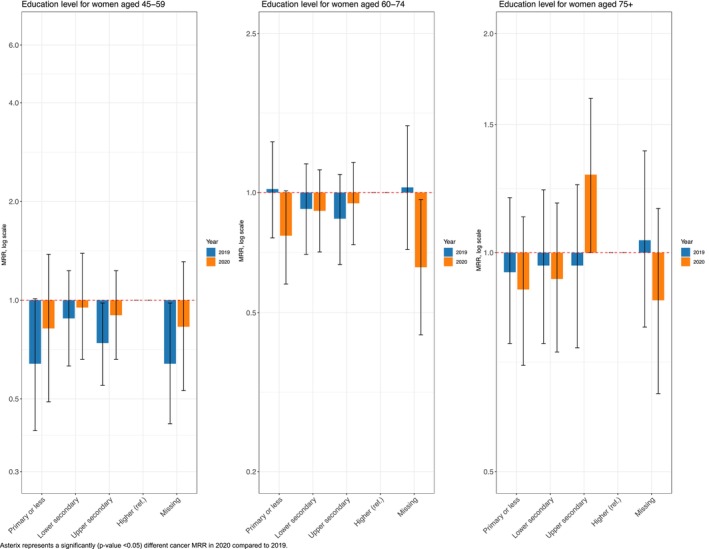
Education‐related cancer mortality rate ratios (MRRs) and 95% CI for breast cancer (C50) in 2020 and 2019, among women aged 45–59, 60–74, and 75+.

Across the three age groups, educational disparities remained unchanged in 2020 compared to those in 2019 (Figure 2). For living arrangements, in contrast, women aged 45–59 living in other households faced a higher relative risk in 2020 than those in 2019 (with women living with a partner being the reference category). Similarly, women aged 60–74 with low income (compared to those with high income) had an increased relative risk in 2020 compared to those in 2019 (see Data S1).

#### 3.2.4 Colorectal Cancer

**FIGURE 3:**
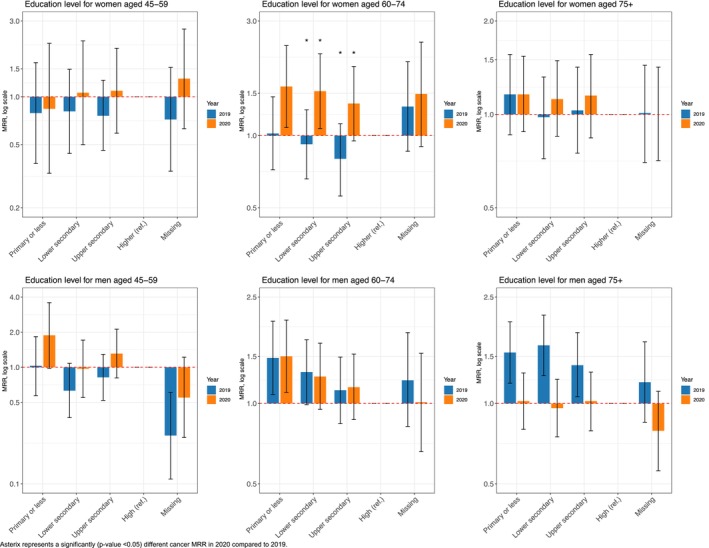
Education‐related cancer mortality rate ratios (MRRs) and 95% CI for colorectal cancer (C18–C20) in 2020 and 2019, among the 45–59, 60–74, and 75+ age groups, by sex.

Women aged 60–74 years with lower secondary education had a 53% higher relative risk of cancer mortality in 2020 compared to those in 2019, relative to women with higher education (MRR in 2020: 1.53; 95% CI: 1.07, 2.19) (Figure 3).

#### 3.2.5 Lung Cancer

**FIGURE 4:**
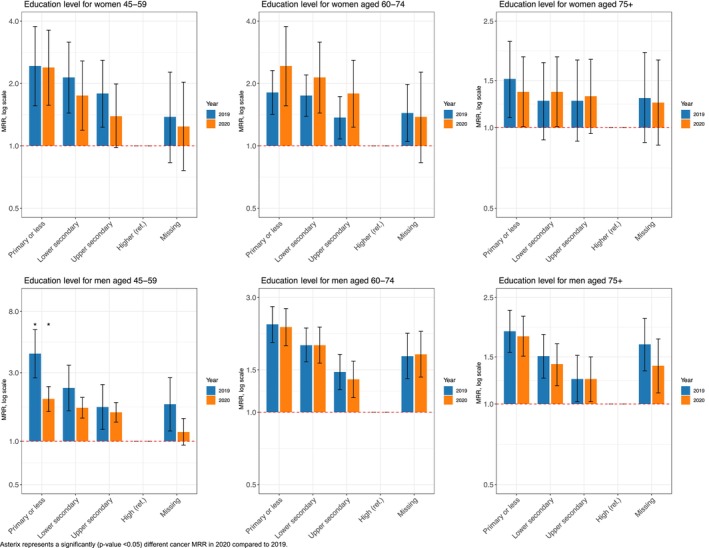
Education‐related cancer mortality rate ratios (MRRs) and 95% CI for lung cancer (C33–C34) in 2020 and 2019, among the 45–59, 60–74, and 75+ age groups, by sex.

Men aged 45–59 years with primary or less education had nearly a two‐fold increase in relative risk in 2020 compared to those in 2019, relative to men with high education (MRR in 2020: 1.97; 95% CI: 1.61, 2.40) (Figure 4). In contrast, older men living in other households faced an increased relative risk in 2020 compared to those in 2019, relative to those living with a partner (see Data S1).

#### 3.2.6 Pancreatic Cancer

**FIGURE 5:**
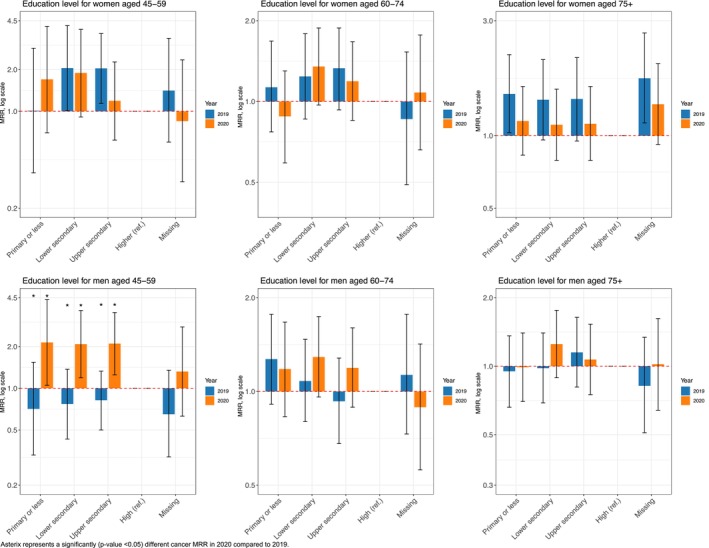
Education‐related cancer mortality rate ratios (MRRs) and 95% CI for pancreatic cancer (C25) in 2020 and 2019, among the 45–59, 60–74, and 75+ age groups, by sex.

In 2020, men aged 45–59 years with less than higher education (compared to high‐educated men) had double the relative risk (MRR_Primary or less_ in 2020: 2.14; 95% CI: 1.05, 4.36; MRR_Lower secondary_ in 2020: 2.08; 95% CI: 1.19, 3.64; MRR_Upper secondary_ in 2020: 2.10; 95% CI: 1.25, 3.52) compared to those in 2019 (Figure 5).

#### 3.2.7 Prostate Cancer

**FIGURE 6:**
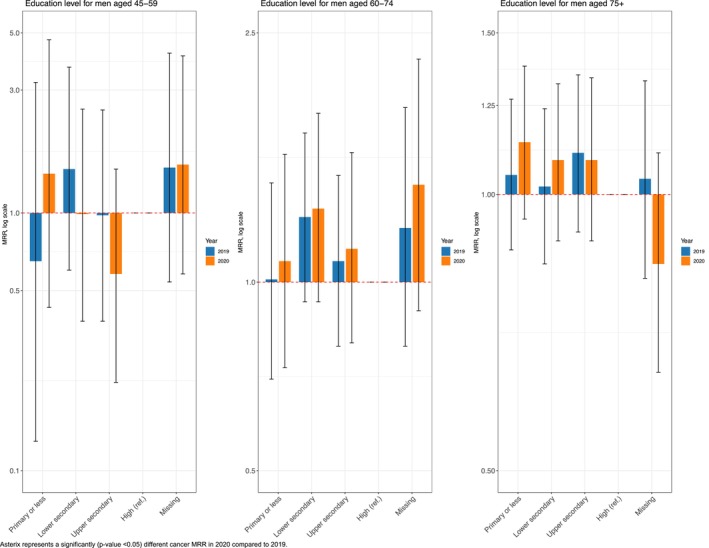
Education‐related cancer mortality rate ratios (MRRs) and 95% CI for prostate cancer (C61) in 2020 and 2019, among men aged 45–59, 60–74, and 75+.

Across the three age groups, no significant differences in educational disparities in prostate cancer mortality were observed in 2020 compared to those in 2019 (Figure 6).

## 4 Discussion

This paper aimed to evaluate changes in overall and site‐specific cancer mortality between 2019 and 2020, and to assess shifts in social inequalities in cancer mortality. In addressing our first research question, we observed significant declines in cancer mortality mainly among men and women aged 75 and over, for both overall and site‐specific cancers. The ongoing decrease in cancer mortality over recent decades might partly explain the drop seen in 2020. For example, lung cancer mortality in men fell by approximately 40% from 2010 to 2020, which represents an average annual reduction of 4% [26]. However, this trend does not fully account for the sharp 21% decline in lung cancer mortality observed in 2020 compared to that in 2019, indicating that other factors were also at play during that year. Similarly, the decreases in mortality observed in other cancer types during the same period could not be fully explained by the long‐term trends, suggesting that additional factors contributed to the decline in cancer mortality rates. Studies in the United States and Mexico reported similar reductions in cancer‐related deaths, likely influenced by COVID‐19 being coded as the underlying COD, in line with WHO guidelines [10, 11]. These guidelines recommended prioritizing COVID‐19 as the underlying cause even when patients were affected with another serious disease such as cancer, cardiovascular disease, chronic respiratory disease, or diabetes [12], possibly leading to underreported cancer mortality in 2020. In contrast, one large US study found that cancer mortality increased when multiple causes of death were considered, particularly for prostate and hematologic cancers [8]. Another US study observed that coding changes during the pandemic led to lower underlying‐cause cancer mortality but an increase in multiple‐cause cancer deaths, especially in the first wave [9]. Even after adjusting for these changes, only a modest 3% rise in cancer deaths was observed, suggesting that COVID‐19 as a competing risk may have influenced mortality trends [9]. This suggests that studies focusing only on underlying causes may miss the broader pandemic impact on cancer mortality. COVID‐19 as a competing risk could affect mortality reporting since both diseases can interact to worsen outcomes; for example, severe COVID‐19 can cause inflammation and immune suppression that aggravates cancer, whereas cancer treatments may increase COVID‐19 severity by weakening immunity [27, 28, 29]. Consequently, selecting one condition as the primary COD often precludes the other from being recorded as such, affecting reported cancer mortality rates. Additionally, lockdowns and social distancing may have reduced cancer patient's exposure to infectious diseases, potentially lowering their risk of death [30]. In addressing our second research question regarding shifts in social inequalities in cancer mortality in 2020 compared with those in 2019, our results showed that, overall, the COVID‐19 pandemic did not considerably change the socioeconomic patterns of cancer mortality. This shows the tenacity of social differences in cancer mortality, which are unlikely to be shifted in less than a year [31]. When examining the five selected cancer types, we found that for more preventable cancers, such as colorectal, lung, and pancreatic cancers, individuals with lower education continued to face higher mortality risks than their higher educated counterparts. This was particularly evident among men aged 45–59 and women aged 60–74 who showed increased relative mortality in 2020 compared to those in 2019 [32]. These disparities were less pronounced among the oldest individuals, while no significant educational differences were observed for less preventable cancers, such as prostate cancer in men or breast cancer in women [32]. Several conclusions arise from our analysis. First, while the COVID‐19 pandemic introduced significant challenges to healthcare systems, the pre‐existing patterns of educational inequalities in cancer mortality largely persisted through 2020. These persistent disparities highlight that longstanding differences in access to healthcare, health literacy, and preventive care did not shift substantially during the crisis [33]. Second, our findings indicate that the efforts by policymakers and healthcare systems to maintain cancer care during the pandemic were effective in preventing an exacerbation of relative cancer mortality inequalities. Finally, to achieve greater equity in healthcare outcomes, it remains essential to continue addressing these entrenched educational inequalities. This involves improving access to healthcare, enhancing health literacy, and strengthening preventive care initiatives, particularly for socioeconomically disadvantaged groups. Sustained and strategic policy efforts are crucial for narrowing these social disparities over the long term, beyond the immediate context of a health crisis.

The study's major strength lies in its use of a high‐quality, nationwide individually linked COD dataset. By analyzing various cancer types, our study provides nuanced insights into how cancer mortality was impacted by the pandemic. Furthermore, we conducted a sensitivity analysis to assess whether the observed decrease in cancer mortality in 2020 compared to that in 2019 was due to a misattribution of COVID‐19 as the underlying COD for individuals with cancer [11, 31]. The sensitivity analysis showed similar results to our initial results, suggesting that the decrease in cancer mortality may not be solely due to misattribution but could also reflect additional factors, such as competing mortality risks between COVID‐19 and cancer. Nevertheless, uncertainties remain about the consistency in how death certificates were coded during this period, as coding practices may have been less complete than in the pre‐COVID period, with cancer potentially not always included in additional COD codes. A limitation relates to the relatively limited number of cancer deaths in specific subpopulations and for specific cancer sites. Additionally, our dataset only included mortality data, lacking information on other cancer outcomes such as cancer incidence or survival, thus limiting our understanding of the pandemic's full impact on cancer outcomes. Our study's focus on cancer‐specific mortality may be complicated by the competing risk dynamic introduced by COVID‐19, particularly as it disproportionately affected socioeconomically disadvantaged groups with higher COVID‐19 mortality [34]. This may result in lower recorded cancer mortality in these groups, potentially reflecting a shift toward COVID‐19 deaths rather than genuine improvements in cancer outcomes [17]. Such shifts could obscure persistent or worsening inequalities. Without considering total and COVID‐19 mortality, reductions in cancer mortality might be misinterpreted. We recognize that a simultaneous analysis of all‐cause mortality would provide deeper insight into these dynamics and better contextualize social inequalities. Future research should aim to incorporate this broader approach. Additionally, we categorized income groups based on the overall population distribution to enable cross‐age group comparisons. However, we acknowledge that this approach may be limited by the non‐linear trajectory of income over the life cycle, which typically increases in early adulthood and declines in older age. Consequently, this could lead to a skewed representation within income deciles, particularly among older adults, with fewer individuals appearing in higher income brackets. While an age‐specific income classification might offer more detailed insights into within‐group inequalities, it would impede cross‐group comparisons. This limitation should be considered in future studies to better capture socioeconomic disparities in older age groups. Moreover, it is important to note that our results are based on a short time frame (March 2020 until December 2020), during which strong social restrictions were implemented. Future research should investigate the evolution of cancer deaths beyond 2020 to ascertain whether the observed declining trend persists or if excess cancer mortality emerges in the long run. Once data become available, we plan to extend this analysis to include more recent years to gain a clearer picture of these trends over time. Delays in cancer diagnoses and treatment during the first stages of the pandemic are likely to be reflected in cancer mortality in the longer term, potentially causing even greater health and economic burden [7, 35]. Extending the analysis to 2021 would also provide valuable insights, as the introduction of vaccinations and shifts in COVID‐19 mortality may alter the competing risk dynamics that affected cancer mortality in 2020. Furthermore, it would be interesting to examine how social inequalities in cancer mortality changed after the reimplementation of social restrictions in 2021, as well as how they evolved during periods of returning to normalcy. Future research could further delve into the coding practices for COD during the COVID‐19 pandemic in Belgium, extending the analysis over a longer time frame to better understand these trends and implications.

## 5 Conclusions

This study highlights the complexities in interpreting cancer mortality changes in 2020 compared to those in 2019, noting that reductions in reported cancer deaths were likely influenced by the prioritization of COVID‐19 as the underlying COD, rather than true declines in cancer mortality. Findings suggest that focusing solely on underlying causes may obscure the broader impact of the pandemic, as COVID‐19 and cancer often interact as competing risks that worsen outcomes. Future research should therefore incorporate overall mortality, multiple COD, and competing risks to better capture the pandemic's full effect on cancer mortality.

Our analysis of social inequalities reveals persistent disparities: individuals with lower education continued to experience higher mortality from preventable cancers, with little change during the pandemic. These ingrained disparities underscore the importance of sustained collaboration between policymakers and healthcare systems to improve healthcare access, preventive care, and health literacy—efforts that are essential for achieving more equitable health outcomes and strengthening preparedness for future public health crises.

Future studies should extend these investigations across a broader time frame, including 2021 and beyond, to validate our results and assess the potential longer term impact of the pandemic on cancer mortality and associated social disparities. Such research will be critical in guiding targeted interventions for future public health crises.

## Author Contributions

**Yasmine Khan:** conceptualization (lead), data curation (lead), formal analysis (lead), investigation (lead), methodology (lead), writing – original draft (lead), writing – review and editing (lead). **Laura Van den Borre:** data curation (supporting), formal analysis (supporting), methodology (supporting), supervision (supporting), validation (supporting), writing – review and editing (supporting). **Delphine De Smedt:** supervision (supporting), validation (supporting), writing – review and editing (supporting). **Nick Verhaeghe:** writing – review and editing (supporting). **Brecht Devleesschauwer:** funding acquisition (lead), supervision (supporting), validation (supporting), writing – review and editing (supporting). **Patrick Deboosere:** supervision (supporting), writing – review and editing (supporting). **Katrien Vanthomme:** conceptualization (supporting), data curation (supporting), formal analysis (supporting), methodology (supporting), supervision (supporting), validation (supporting), writing – review and editing (supporting). **Sylvie Gadeyne:** conceptualization (supporting), methodology (supporting), supervision (supporting), validation (supporting), writing – review and editing (supporting).

## Ethics Statement

The authors have nothing to report.

## Conflicts of Interest

The authors declare no conflicts of interest.

## Supporting information


Data S1.

## Data Availability

The data that support the findings of this study are available in Data S1 of this article.
